# Regulatory mechanisms and metabolic changes of miRNA during leaf color change in the bud mutation branches of *Acer pictum subsp*. *mono*

**DOI:** 10.3389/fpls.2022.1047452

**Published:** 2023-01-12

**Authors:** Baoli Lin, He Ma, Kezhong Zhang, Jinteng Cui

**Affiliations:** ^1^ College of Landscape Architecture, Beijing University of Agriculture, Beijing, China; ^2^ Laboratory of Urban and Rural Ecological Environment, Beijing University of Agriculture, Beijing, China

**Keywords:** *Acer pictum subsp.mono*, leaf color, differential metabolite, microRNA, regulatory network

## Abstract

*Acer pictum subsp. mono* is a colorful tree species with considerable ornamental and economic value. However, little is known about the metabolism and regulatory mechanism of leaf color change in *A. p. subsp. mono*. To reveal the molecular mechanism of leaf color change in *A. p. subsp. mono*, the present study examined the bud mutation branches and compared the metabolites of the red leaves (AR) of the bud mutation branches of *A. p. subsp. mono* with those of the green leaves (AG) of the wild-type branches. It was found that the chlorophyll and carotenoids content of the red leaves decreased significantly, while anthocyanins, and various antioxidant enzymes increased significantly compared with the green leaves. The glycosides cyanidin, pelargonidin, malvidin, petunidin, delphinidin, and peonidin were detected in AR by liquid chromatography-mass spectrometry. The cyanidin glycosides increased, and cyanidin 3-O-glycoside was significantly upregulated. We analyzed the transcriptome and small RNA of *A. p. subsp. mono* leaves and detected 4061 differentially expressed mRNAs and 116 differentially expressed miRNAs. Through miRNA-mRNA association analysis, five differentially expressed modules were found; one miRNA targeted three genes, and four miRNAs targeted a single gene. Among them, miR160b, miR6300, and miR396g were found to be the key miRNAs regulating stable anthocyanin accumulation in *A. p. subsp. mono* leaves. By revealing the physiological response of leaf color change and the molecular regulatory mechanism of the miRNA, this study provides new insight into the molecular regulatory mechanism of leaf color change, thereby offering a foundation for future studies.

## 1 Introduction

*Acer pictum subsp. mono* is a perennial deciduous tree of the Acer genus. Distributed widely in northeastern, northern, and southern China, it enjoys humid conditions and fertile acidic soil. Its shape and red leaves in autumn have high ornamental value, rendering it an excellent landscape tree species that is widely used in landscaping. It is also an important autumnal species and native tree species in northern China (Editorial Board of Tree Records of China, 2005).

For ornamental plants, the color of the leaves is a key feature contributing to their ornamental value (Tang et al., 2020). Although the phenomenon of leaf color change attracts great attention every year, its mechanisms and reasons remain unclear and controversial (Duan et al., 2014). However, according to previous studies, the color change of plant leaves is mainly determined by genetic factors but is also affected by environmental factors, which jointly regulate the synthesis and metabolism of plants, thus causing changes in photosynthetic pigments, anthocyanins, physiological and biochemical indexes, and leaf structure, ultimately resulting in the color of the leaves changing (Li et al., 2017). For instance, the leaves of Cotinus coggygria are bright red because of their high anthocyanin content (Ge et al., 2011). The main reason why Malus pumila turns red is that carotenoids and anthocyanins gradually accumulate, while chlorophyll is degraded (Saure, 1990). However, in Betula platyphylla, the expression level of genes related to chlorophyll metabolism increased, and the leaves gradually turned yellow with the intensification of chlorophyll degradation (Sinkkonen et al., 2012). It is thought that soluble sugar is the energy substance and trigger for anthocyanin synthesis (Schaberg et al., 2003), which acts as a signaling molecule in the process of anthocyanin synthesis and activates some enzymes in anthocyanin synthesis (Vitrac et al., 2000). Carbohydrates can also regulate the expression of genes related to plant metabolic functions, including the expression of anthocyanins in plant tissues (Dube et al., 1993).

MicroRNAs (miRNAs) are endogenous, single-stranded, small, non-coding RNAs (Hua et al., 2019). Studies have shown that miRNAs can regulate cell proliferation, differentiation, and senescence by regulating post-transcriptional genes, thereby playing a variety of roles in plant life. miRNA participates in a variety of physiological processes such as plant growth, development, apoptosis, and stress resistance (Zhang et al., 2013; Yang et al., 2019; Jatan et al., 2019). The process of plant leaf color transition depends on the expression of many genes with transcriptional and post-transcriptional regulation mechanisms, which mainly occurs at the transcriptional level (Zheng et al., 2019). In Arabidopsis thaliana, the regulatory modules of miR156-AtSPL9 and miR858-AtMYB2 have been confirmed. SPL9 is an inhibitor of anthocyanin synthesis in senescent plant cells, and miR156 negatively regulates SPL9 in young plant cells, making downstream transcription factors activate anthocyanin production (Gou et al., 2011; Zhang et al., 2021; Guan et al., 2014). In Chinese Rosa chinensis, the miR156-RcSPL9 regulatory module was also found to affect anthocyanin synthesis in the petals (Raymond et al., 2018). In Diospyros kaki, the miR397-DkLAC2 regulatory module was found to influence the biosynthesis of proanthocyanidins (Zaman et al., 2022). Metabolites are the end products of the response of plants to genetic or environmental (Fiehn, 2002). Metabonomics is closest to the phenotype and can reflect the physiological state of organisms more directly and accurately than proteomics or transcriptomics. Targeted metabolomics is a new metabonomics detection technology that combines the advantages of high-throughput non-targeted metabolomics with the advantages of the high accuracy and high sensitivity of targeted metabolomics (Wang et al., 2016). A comprehensive analysis of the transcriptome and metabolome can also provide information about the relationship between genes and metabolites and the potential complex molecular regulatory networks (Yan et al., 2018).

**Figure 1:**
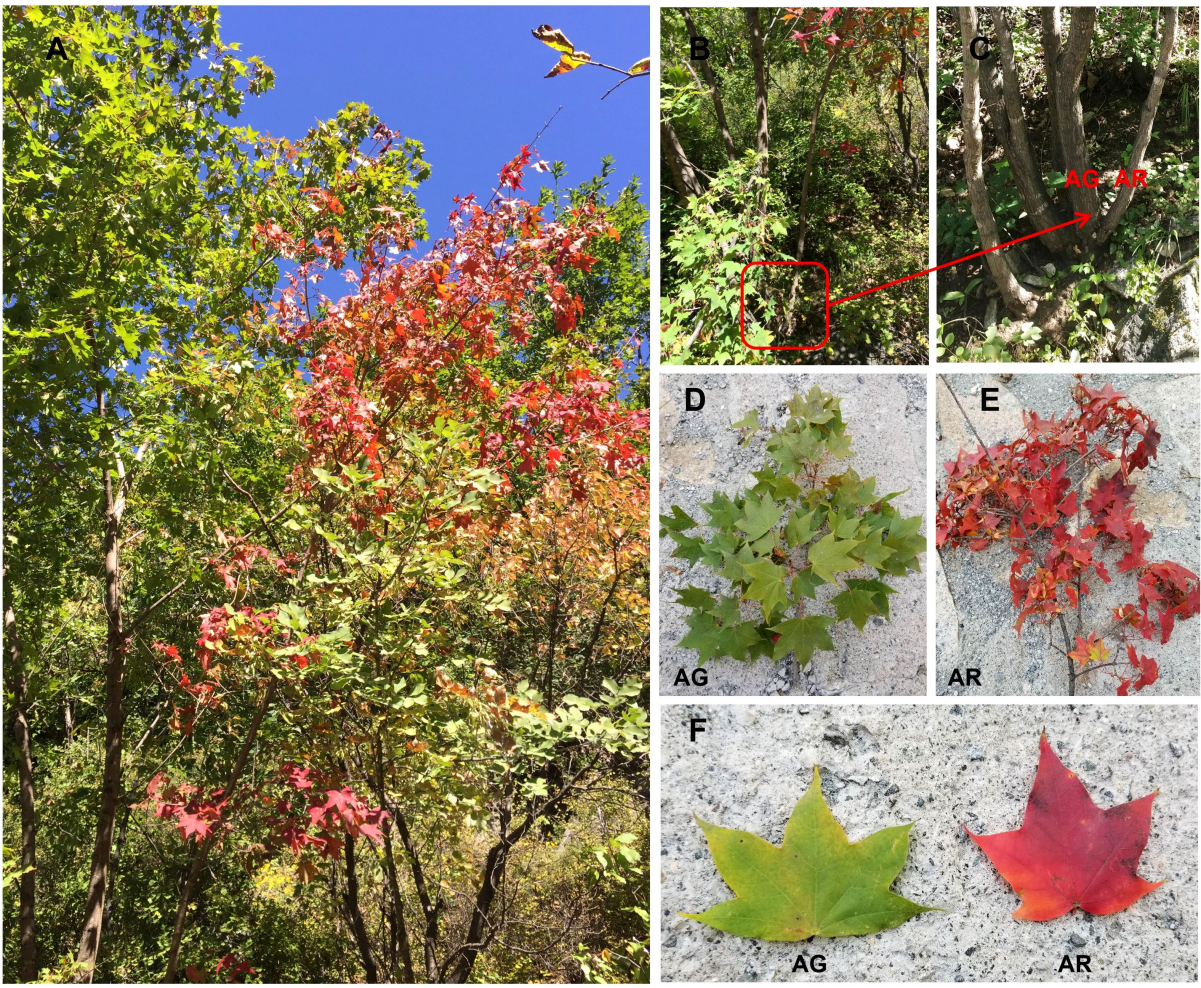
*Acer p. subsp*. *mono* with bud mutation branches found in the wild. **(A)** *Acer p. subsp*. *mono* with bud mutation branches in autumn. **(B, C)** are wild-type branches (AG) and bud mutation branches (AR) of *A. p. subsp. mono* in autumn. **(D–F)** Green leaves of wild-type branches (AG) and red leaves of bud mutation branches (AR) in autumn.

At present, many studies have focused on the regulatory mechanism of leaf color change in Acer plants with respect to physiology (Stokowy et al., 2014), but few studies have investigated the miRNA, transcription factors, and the metabolome. To explore the molecular regulatory mechanism of the color change process of *A. p. subsp. mono* leaves, we obtained bud mutation branches from Yueliangan, Huairou District, Beijing, which turned red 15–20 days earlier than the leaves of the wild plants(as shown in **Figure 1**). After three years of continuous observation, the characteristics of the bud mutation branches were found to be stable. To better understand the regulatory mechanisms of miRNAs-mRNA and the change law of small-molecule metabolites in the leaves during color change in the buds, we analyzed the mRNA and miRNA sequences of *A. p. subsp. mono* leaves during the color transition (Lu et al., 2008). We used high-throughput sequencing and bioinformatics tools to identify known miRNAs and new miRNAs and their target genes, and analyzed the small-molecule metabolites of *A. p. subsp. mono* leaves by liquid chromatography-mass spectrometry (LC-MS). The purpose of this paper was to analyze the changes in mRNA, miRNA, and metabolites in the bud-mutated branches and leaves of wild *A. p. subsp. mono* and to reveal the associated mechanisms.

## 2 Materials and methods

### 2.1 Plant materials

In October 2017, we discovered a wild *A. p. subsp. mono* in Yueliangan, Huairou District, Beijing, which had a bud mutation branch. The leaves of the bud-changed branch turned red 15–20 days earlier than those of the wild-type branches. After continuous observation for three years, the various characteristics of the bud mutation branches remained stable. In August 2019, which is summer in Beijing, the green leaves of wild *A. p. subsp. mono* were collected. We collected the green leaves of the bud mutation branches of wild *Acer pictum subsp.mono.*and named S. In October 2019, in autumn in Beijing, the leaves of wild *A. p. subsp. mono* were in the early stage of discoloration. At this time, the leaves on the bud branches had turned red, while the leaves of wild-type branches were still green. Healthy red leaves were collected from the bud mutation branches, named AR, and healthy green leaves from the wild-type branches, named AG (as shown in **Figure 1**), were also collected. These leaves were stripped of their petioles and receptacles, leaving only the blades. The blades were removed, placed on dry ice, transported back to the laboratory, and stored in an ultra-low temperature refrigerator at −80°C.

### 2.2 Physiological analysis

The leaves of the S group, AG group, and AR group of *A. p. subsp. mono* were measured for Chl a content, Chl b content, carotenoid content, PAL activity, PPO activity, SOD activity, POD activity, CAT activity, and soluble sugar content. Using ethanol extraction colorimetry, the Chl a, Chl b, and carotenoid content were measured (Yan et al., 2018). The content of anthocyanins was measured by hydrochloric acid ethanol extraction colorimetry (Pietrini and Massacci, 1998). The PAL activity and PPO activity were measured using the modified method of Ravichandran and Parthiban (Ravichandran and Parthiban, 1998). The SOD activity was determined by the nitrogen blue tetrazole method (Zhang et al., 1995). The activity of oxidase (POD) and catalase (CAT) was measured by Braber’s improved method (Braber, 1980). Anthrone colorimetry was used to determine the soluble sugar content (Ebell, 1969). All data were analyzed using Student’s t-test (SPSS 17.0) with five replicates.

### 2.3 Metabolome analysis

The leaves of the AG group and AR group of *A. p. subsp. mono* were used as experimental materials, and each group was set with 3 replicates. Extraction and LC-MS/MS analysis of metabolites from leaves of *A. p. subsp. mono* were carried out in Novogene, Beijing, according to their standard procedure (https://cn.novogene.com/tech ) and the description of Luo et al. (Want et al., 2013; Luo et al., 2015). Detection of the experimental samples using MRM was based on Novogene in-house database. Q3 (daughter) was used for the quantification. Q1 (parent ion), Q3,retention time, declustering potential, and collision energy were used for metabolite identification. Data files generated by HPLC-MS/MS were processed with SCIEX OS(version 1.4) to integrate and correct the peaks. Before the data analysis, a quality control (QC) analysis was performed to confirm the reliability of the data (Zhuang et al., 2019). MetaX (Wen et al., 2017) software was used to analyze the data by PCA principal component analysis, which reflected the overall metabolic differences among samples in each group and the variance between samples in each group. The KEGG database (http://www.genome.jp/kegg/ ) was used to annotate the pathways of the detected metabolites. Set the threshold value to VIP > 1.0, difference multiple FC > 1.5 or FC < 0.667 and P-value < 0.05 to screen out differential metabolites (Sreekumar et al., 2009; Haspel et al., 2014; Heischmann et al., 2016), and draw volcano map based on Log2 (FC) and-log10 (P-value) of metabolites to show the overall distribution of differential metabolites. The Top20 KEGG pathway with significant enrichment of differential metabolites was screened and the bubble map of the KEGG pathway was plotted.

### 2.4 Small RNA analysis

Extracted by EASYspin Plant microRNA Kit (Aidlab, Beijing Aidlab Biotechnology Co., Ltd., China)AG phase, AR phaseAdj.RNA, using 1% agarose gel electrophoresis andNanodropNC 8000The concentration and purity of RNA were detected. Sequencing and assembly completed by Novogene Co., Ltd. The clean reads were applied for small RNA analysis. The number of clean reads was counted while the sequence length is more than 18 NT and less than 30 NT. The identical sequence in a single sample was deduplicated and the sequence abundance was counted to obtain unique reads for subsequence analysis sets AG and AR to repeat three times respectively. Using AASRA (Tang et al., 2021) to filter the sequence data; Sequences not matched with the miRBase database were used to predict new miRNA by miREvo (Wen et al., 2012) and mirdeep2 (Friedlander et al., 2012) software. Using TPM (‘t Hoen et al., 2008) software to quantify the expression quantity, using DESeq2 (Fahlgren et al., 2007) to analyze the differential expression of miRNAs, and using padj < 0.05 as the threshold, screening the differentially expressed miRNAs, and cluster analysis of the differential miRNAs. Target Finder (Ma et al., 2020) software was used to predict the target genes of differentially expressed miRNAs, and the target genes of differentially expressed miRNA were obtained. KEGG enrichment analysis was carried out on the predicted target genes of differentially expressed miRNA.

### 2.5 Transcriptome analysis

Use EASYspin Plus Complex Plant RNA Kit(Beijing Aidlab Biotechnology Co., Ltd., China) Extracting AG and AR leaves a total RNA 3 replicate per sample. The purity and concentration of RNA were evaluated using 1% agarose gel electrophoresis and NanoDrop NC 8000 spectrophotometer (NanoDrop, Thermo Scientific, Germany), and then the integrity of RNA was evaluated using Agilent 2100 Bioanalyzer (Agilent Technologies, USA). Sequencing and assembly of RNA are composed of completed by Novogene Co., Ltd. The genome of Acer truncatum https://doi.org/10.6084/m9.figshare.12986237 . v2. (Love et al., 2014) was used for reference analysis, DESeq2 software (Fahlgren et al., 2007) was used, and log2 (FoldChange) ≥ 1 and padj were used ≤ 0.05 for statistical analysis. A volcanic map was used to show the distribution of differential genes in each combination. KEGG database (http://www.genome.jp/kegg/) was used to annotate the pathway of DEGs, and differential clustering analysis was carried out for differential genes.

### 2.6 miRNA-mRNA association analysis

According to AG and AR period miRNA and mRNA data difference relationship, combined with miRNA and mRNA targeting relationship and Pearson coefficient value, the regulatory network diagram of miRNA and target gene was drawn to show their interaction relationship. Choose the correlation coefficient above 0.6 as the significant correlation targeting relationship.

### 2.7 Construction of phylogenetic tree

The amino acid sequences of three key genes ApUFGT, ApSUS and ApUGP2 in the leaves of *A. p. subsp. mono* were searched for homologous sequences of Arabidopsis thaliana, Oryza sativa, Nicotiana tabacum, Glycine max, Prunus persica, and Malus pumila through BLAST online at https://phytozome-next.jgi.doe.gov/. The amino acid sequences of ApUFGT, ApSUS and ApUGP2 were compared with those of Arabidopsis thaliana,Oryza sativa, Nicotiana tabacum, Glycine max, Prunus persica, and Malus pumila by Mega 7.0. The phylogenetic tree was constructed by JJT + G model with Bootstrap 1000 and neighbor-joining method. The successfully constructed phylogenetic tree is displayed and annotated using iTOL software (https://iTOL : Login (embl.de) .

### 2.8 Quantitative RT-PCR analysis

Through real-time fluorescence quantitative analysis, it is verified that miR160b, miR396g, miR6300, ApUFGT, ApSUS, and ApUGP2. With SMART MMLV Reverse Transcriptase (Takara Bio Inc., China) Reverse transcription of RNA; Reverse transcription reaction of miRNA was performed with Green miRNA First-Strand cDNA Synthesis SupperMix (TransGene, China).According to the screened miRNA and target gene sequence, fluorescence quantitative PCR primers were designed (**Table S2 and S3**). 18S and U6 were selected as target gene and miRNA control, respectively. RT-PCR was also carried out on a iQ5 RT-PCR System (Bio-Rad, USA) using an 2× TransStart Top Green qPCR SuperMix (TransGene, China) andGreen miRNA Two-Step qRT-PCR SupperMix(TransGene, China). All the above are according to the manufacturer’s instructions. Amplification actions were performed as follows: 94 °C 5s, 60 °C 15s, and 72 °C 10s.; 45 Allreactions were carried out in triplicate. The quantitative changes were evaluated by the relative quantitative comparative Ct method (2-ΔΔCt). All quantitative RT-PCR for each gene underwent three biological repetitions and three technical repetitions. The RNA sample used for quantitative RT-PCR is the same as the sample used.

## 3 Results

### 3.1 Analysis of physiological indexes of bud mutation branch leaves and wild-type leaves of *Acer pictum subsp.mono*

**Figure 2:**
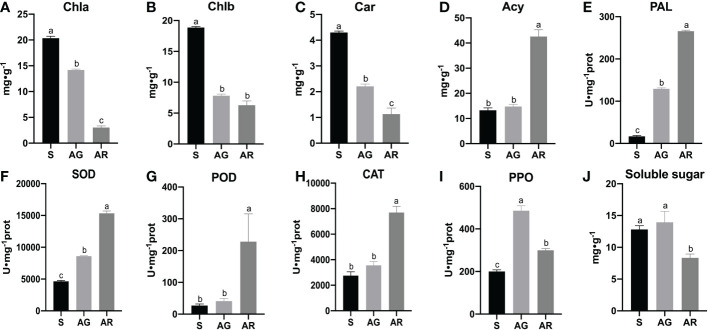
Biochemical indexes of *A. p. subsp. mono* leaves. **(A–J)** show the contents of chlorophyll a, chlorophyll b, carotenoid, anthocyanin, PAL, SOD, POD, CAT, PPO, and soluble sugar in the leaves of *A. p. subsp. mono* at three stages of S, AG, and AR, respectively. S, AG, and AR denote bud mutation branch leaves in summer, wild branch leaves in autumn, and bud mutation branch leaves in autumn, respectively.

We found that there were obvious differences in the physiological indexes between the wild-type leaves (AG) and bud mutation branch leaves (AR) of *A. p. subsp. mono* in autumn(as shown in **Figure 2**). Compared with bud mutation branch leaves of *A. p. subsp. mono* in summer (S), the contents of chlorophyll a (Chl a) chlorophyll b (Chl b),and carotenoids (Car) in AG and AR leaves decreased significantly, and the contents of Chl a, Chl b, and Car in AR leaves decreased most obviously (**Figures 2A, B**). The contents of anthocyanins (Acy) in AG and AR leaves were significantly higher than those in S leaves, and the contents of anthocyanins (Acy) in AR leaves were significantly higher than those in AG leaves (**Figures 2C, D**). The activities of phenylalanine ammonia-lyase (PAL), superoxide dismutase (SOD), peroxidase (POD), and catalase (CAT) in AR leaves were significantly higher than those in AG leaves and S leaves, and the activities of PAL and SOD in AG leaves were significantly higher than those in S leaves (**Figures 2E-H**). The polyphenol oxidase (PPO) activity in AG and AR leaves was significantly higher than that in S leaves (**Figure 2I**). The soluble sugar content of AR leaves (**Figure 2J**) was significantly lower than that of S leaves and AG leaves, but there was no significant difference between S leaves and AG leaves. The activities of phenylalanine ammonia-lyase (PAL) and contents of anthocyanins in AR leaves were significantly higher than those in S leaves and AG leaves, indicating that the leaves of the bud mutation branches had accumulated a large amount of anthocyanins.

Through correlation analysis (**Table 1**), we found that anthocyanins were positively correlated with the activities of SOD, CAT, POD, and PAL; anthocyanins were negatively correlated with chlorophyll a, chlorophyll b, carotenoids, and soluble sugar; carotenoids were positively correlated with chlorophyll a and chlorophyll b; carotenoids were negatively correlated with the activities of SOD, CAT, POD, and PAL; and soluble sugar was negatively correlated with the activities of anthocyanins, SOD, CAT, POD, and PAL.

**Table 1:** Correlation analysis of the leaf biochemical indexes of *A. p. subsp. mono*.

	Chla	Chlb	Car	Acy	SOD	CAT	POD	PAL	PPO	Soluble sugar
Chla	1	0.834**	0.934**	-0.945**	-0.998**	-0.968**	-0.863**	-0.993**	-0.182	0.791*
Chlb		1	0.966**	-0.619*	-0.843**	-0.692**	-0.558*	-0.890**	-0.682**	0.397
Car			1	-0.785**	-0.943**	-0.839**	-0.743**	-0.967**	0.506	0.566*
Acy				1	0.940**	0.982**	0.917**	0.906**	-0.124	-0.890**
SOD					1	0.965**	0.868**	0.995**	0.200	-0.780**
CAT						1	0.913**	0.938**	-0.013	-0.878**
POD							1	0.825**	-0.098	-0.856**
PAL								1	0.288	-0.728**
PPO									1	0.350
Soluble sugar										1

*Represents P < 0.05, ** Represents P < 0.01.

### 3.2 Metabolite analysis of *A. p. subsp. mono* leaves based on targeted metabonomics

**Figure 3:**
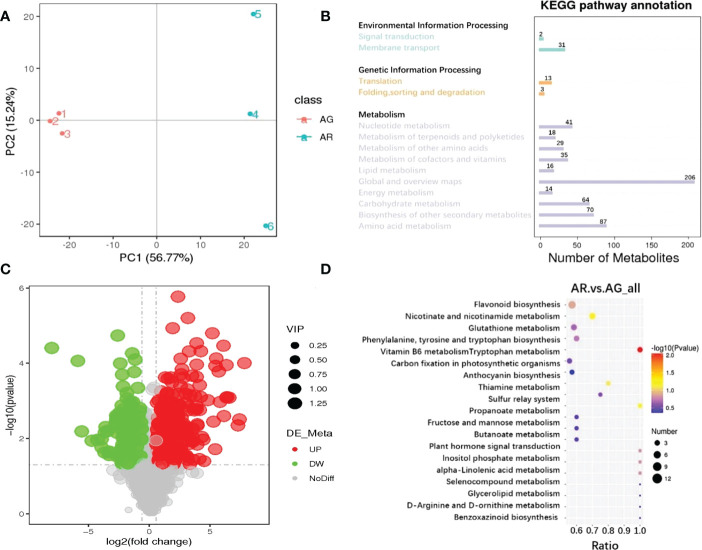
Metabolite analysis of *A p. subsp. mono* leaves based on targeted metabonomics technology. **(A)** PCA analysis of metabolites in the leaves of *A p. subsp. mono.* **(B)** KEGG classification map of metabolites from the leaves of *A p. subsp. mono.* **(C)** Volcanic map of differential metabolites in the leaves of *A p. subsp. mono* (AR vs. AG). **(D)** Enrichment analysis of KEGG (AR vs. AG); differential metabolites in the leaves of *A p. subsp. mono*.

To better elucidate the types and differences in metabolites in the AG and AR leaves of *A. p. subsp. mono*, we used LC-MS technology to conduct a targeted metabolomics analysis of the leaves of AR and AG leaves based on the SCIEX QTRAP ^®^ 6500 + mass spectrometry platform with high sensitivity. Principal component analysis (PCA) was used to observe the metabolite variance between AR and AG leaves. As shown in **Figure 3A**, the cumulative variance contribution rates of PC1 and PC2 were 56.77% and 15.24%, respectively, and the AR and AG leaves could be clearly distinguished by PCA analysis. A total of 1104 metabolites were identified by LC-MS. The Kyoto Encyclopedia of Genes and Genomes (KEGG) classification of the identified metabolites (the first 14 enriched metabolic pathways), (as shown in **Figure 3B**), indicated that the primary distribution levels of the metabolites of AR and AG *A. p. subsp. mono* leaves were mainly in environmental information processing, genetic information processing, and metabolism. The secondary distribution levels in environmental information processing were signal transformation (31 metabolites) and membrane transport (two metabolites). Genetic information processing mainly included translation (13 metabolites), folding, sorting, and degradation (three metabolites). There are 580 metabolites distributed in metabolism, which were mainly concentrated in amino acid metabolism, biosynthesis of other secondary metabolites, carbohydrate metabolism, energy metabolism, global and overview maps, lipid metabolism, metabolism of cofactors and vitamins, metabolism of other amino acids, metabolism of terpenoids and polyketides, and nucleotide metabolism. With variable importance in the projection (VIP) > 1.0, fold-change (FC) > 1.5 or FC < 0.667, and P-value < 0.05 as thresholds, 429 differential metabolites were screened out, of which 291 were upregulated and 138 were downregulated(as shown in **Figure 3C**).

Through KEGG enrichment analysis of the differential metabolites in the leaves of *A. p. subsp. mono* (top-20 metabolic pathways) (as shown in **Figure 3D**), the differential metabolites of AR vs. AG were mainly enriched in vitamin B6 metabolism (100%), nicotinate and nicotinamide metabolism (70%), sulfur relay system (100%), anthocyanin biosynthesis (80%), flavonoid biosynthesis (57%), inositol phosphate metabolism (100%), alpha-linolenic acid metabolism (100%) and plant hormone signal Transduction (100%), phenylalanine, tyrosine and tryptophan biosynthesis (60%), glutathione metabolism (58%), thiamine metabolism (75%), tryptophan metabolism (56%), fructose and mannose metabolism (60%), propanoate metabolism (60%), butanoate metabolism (60%), benzoxazinoid biosynthesis (100%), selenocompound metabolism (100%), D-Arg inline and D-ornithine metabolism (100%), glycerolipid metabolism (100%), and carbon fixation in photosynthetic organizations (57%). Only the vitamin B6 metabolism metabolic pathway was significantly enriched in KEGG (P-value < 0.05)

**Figure 4:**
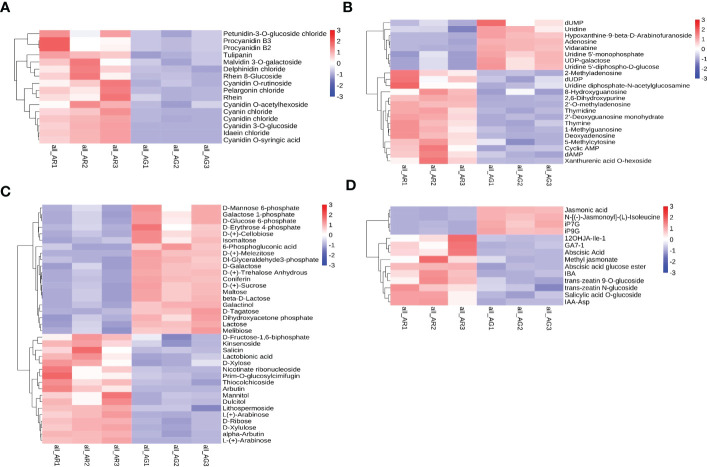
Differential metabolite cluster thermogram of the KEGG pathways. **(A)** Anthocyanins. **(B)** Nucleotides and derivatives. **(C)** Carbohydrates and derivatives. **(D)** Phytohormones.

Through careful screening of the KEGG enrichment results of the differential metabolites in the leaves of *A. p. subsp. mono*, we selected the differential metabolites closely related to the changes in leaf pigments, nucleotides, soluble sugars, and plant hormones. Differential metabolites were divided into four categories: anthocyanins, nucleotides and derivatives, carbohydrates and derivatives, and phytohormones. The relative quantitative values of the differential metabolites were normalized and clustered (**Figure 4**). We screened 16 metabolites belonging to anthocyanins, all of which were significantly upregulated (as shown in **Figure 4**). In AR, cyanidin 3-O-glucoside, cyanidin O-syringic acid, idaein chloride, and cyanidin O-rutinoside were stable anthocyanins, and their contents were upregulated significantly, with corresponding log2FC values of 6.42, 6.37, 6.13, and 4.15, respectively. Cyanidin chloride and pelargonin chloride were unstable anthocyanins and their contents were upregulated significantly. There were 23 different metabolites belonging to nucleotides and derivatives, of which 15 were upregulated and eight were downregulated (**Figure 4B**). Deoxyadenosine, 2 ‘-O-methyladenosine, 2’-deoxyguanosine monohydrate, and 2, 6-dihydroxypurine were significantly upregulated, and the corresponding log2FC values were 5.73, 4.83, 4.53, and 4.46, respectively. The contents of UDP-galactose and UDP-glucose (uridine 5 ‘-diphhospho-D-glucose) were downregulated, and UDP-galactose and UDP-glucose are substrates involved in the process of transforming unstable anthocyanins into stable anthocyanins. UDP-glucose and UDP are involved in sucrose hydrolysis metabolism and anthocyanin anabolism as important substances. There were 37 different metabolites belonging to carbohydrates and derivatives, of which 17 were upregulated and 20 were downregulated (**Figure 4C**). D-Fructose was detected but did not differ significantly between AR vs. AG. There were 14 different phytohormone metabolites, of which 10 were upregulated and four were downregulated (**Figure 4D**). The contents of abscisic acid (ABA) and 3-indolebutyric acid (IBA) were upregulated, and the contents of jasmonic acid (JA) were downregulated. ABA promotes leaf abscission, and the increase in ABA content indicated that the red leaves of the bud mutation branches of *A. p. subsp. mono* had senesced and entered into the abscission period.

### 3.3 Sequencing and identification of known and novel miRNAs

Six small RNA libraries of *A. p. subsp. mono* were constructed using green leaves (AG) and red leaves (AR) from wild-type branches collected in autumn, and each group was divided into three replicates. The minimum values of the Raw tags and Clean tags were 2.7*107 and 2.4*107 and 91% and 99.4% of the total data, respectively. The quality of the sequencing was good. Clean reads were compared to the reference genome and other small RNA databases, including miRBase, Rfam, siRNA, piRNA, and snoRNA, with an average comparison rate of 73.38% (**Table 2**).

**Table 2:** Quality control data statistics and miRNA alignment.

Sample	Raw tag	Clean tag	Q20 of clean tag (%)	Percentage of clean tag (%)	Total tag	Mapped tag	Percentage (%)
AG1	27925033	26107130	99.5	93.49	26107130	19541405	74.85
AG2	29113452	27822202	99.5	95.56	27822202	20761549	74.62
AG3	28555559	26662207	99.4	93.37	26662207	19319194	72.46
AR1	28498582	26411577	99.5	92.68	26411577	20413156	77.29
AR2	27940389	24780967	99.5	88.69	24780967	17964891	72.49
AR3	29108186	27125742	99.5	93.19	27125742	19613748	72.31

Raw tag is the number of original data tags; clean tag is the number of filtered data tags; percentage of clean tags (%) = clean tag count/raw tag count; and clean tag accounts for the percentage of total data.

**Figure 5:**
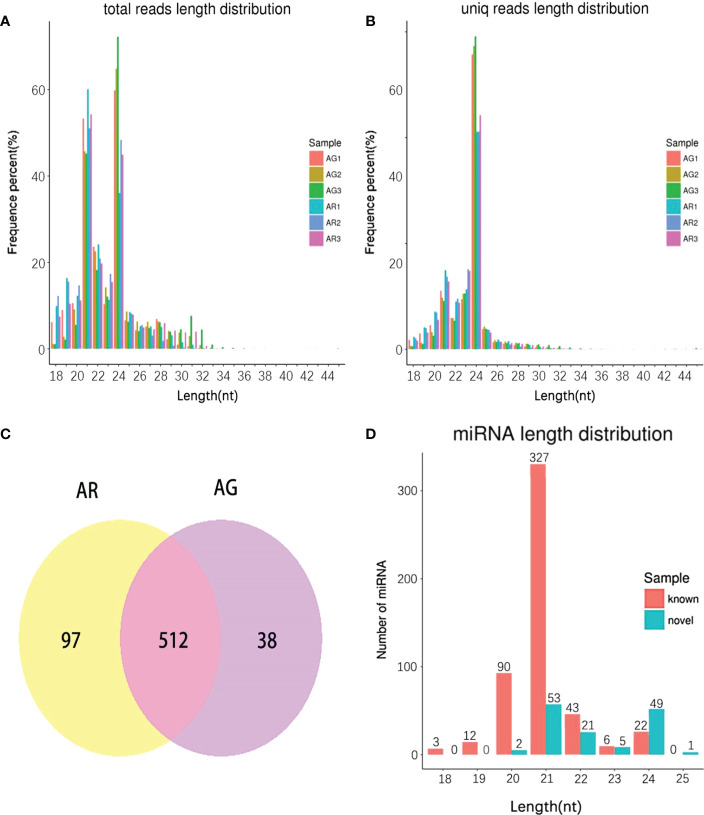
The length distribution of small RNA in *A p. subsp. mono* at different developmental stages and miRNA identity. **(A)** Length distribution of clean reads. **(B)** Length distribution of unique reads. **(C)** Venn Diagram. **(D)** Length distribution of known miRNA and novel miRNA.

The length of 24 nt was the most abundant class among the clean and unique reads (**Figures 5A, B**) produced by high-throughput sequencing in the six small RNA libraries of *A. p. subsp. mono*. A total of 647 miRNAs were detected, of which 97 miRNAs were detected only in AR and 38 miRNAs were detected only in AG (**Figure 5C**). A total of 510 known miRNA were found. These known miRNAs were divided into 77 families, of which MIR156 was the largest family with 53 members, followed by the MIR396 family with 46 members (**Table S1**). After the mature known miRNA was determined, the remaining sequences that did not match the database were formed by complementary pairing based on the precursor structure of the miRNA, and novel miRNAs were predicted by miRNA (Evers et al., 2015) software. We predicted 137 novel miRNAs. The length of the known miRNAs ranged from 18 to 24 nt, and that of the novel miRNAs ranged from 20 to 25 nt (**Figure 5D**). Most mature sequences in the known miRNAs were concentrated at 21 nt, which is consistent with a previous report (Zhao et al., 2020). However, most of the novel miRNAs were scattered, with 21 nt and 24 nt being more common.

### 3.4 Enrichment analysis of differentially expressed miRNAs and mRNAs

**Figure 6:**
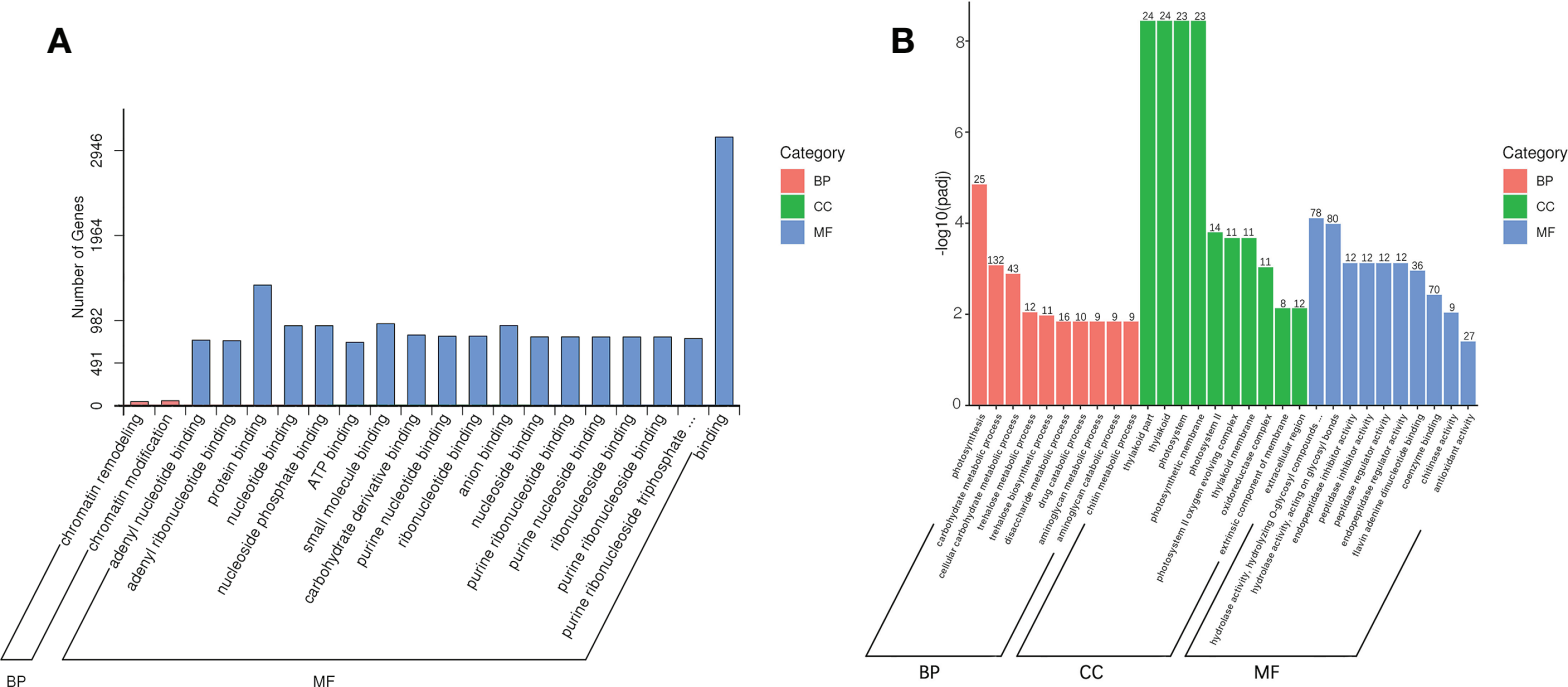
Enrichment analysis of differentially expressed miRNAs and mRNAs. **(A)** GO enrichment analysis of differentially expressed miRNAs target genes. **(B)** GO enrichment analysis of differentially expressed mRNAs.

With reference to the genome of Acer truncatum, we analyzed the transcriptome of AG and AR of A. truncatum. Using DESeq2 software, the differential genes were screened with log2 (fold-change) > 1 and padj < 0.05 as thresholds. Comparing AR with AG, 2513 mRNAs were upregulated and 1548 mRNAs downregulated (**Figure S1**). At the same time, we analyzed the differential expression of miRNAs between AG and AR of *A. p. subsp. mono*. Comparing AR vs. AG with padj < 0.05, 75 miRNAs were upregulated, while the expression level of 41 miRNAs was downregulated (**Figure S2**). To better understand the function of the miRNAs, we referred to the genome of A. truncatum and used TargetFinder to predict the target genes of the miRNA, with a total of 72,444 target genes predicted. After AG and AR differentially expressed miRNAs were obtained, we carried out Gene Ontology (GO) enrichment analysis for each set of differentially expressed miRNA target genes according to the corresponding relationship between the miRNA and its target genes (**Figure 6A**, P < 0.05). In the biological process (BP) category, chromatin remodeling biologic process (GO: 0006338) and chromatin modification biologic process (GO: 0016568) were significantly enriched. In the molecular function (MF) category, 18 GO terms were significantly enriched, most of which were related to ribonucleotide binding. To better understand the functional classification of the differentially expressed mRNAs, we carried out a GO enrichment analysis on differentially expressed mRNAs between AR and AG. The top-10 BP, MF and cellular component (CC) terms were plotted as a GO enrichment map (**Figure 6B**, padj < 0.05). In BP, GO terms related to photosynthesis and glucose metabolism were significantly enriched, such as photosynthesis (GO: 0015979), carbohydrate metabolic process (GO: 0005975), and disaccharide metabolic process (GO: 0005984). In CC, GO terms related to photosynthesis were enriched significantly, such as photosystem (GO: 0009521), photosynthetic membrane (GO: 0034357), and photosystem II (GO: 0009523). In MF, GO terms related to hydrolyzed glycosyl groups were enriched significantly, such as hydrolase activity, hydrolyzing O-glycosyl compounds (GO: 0004553), and hydrolase activity, acting on glycosyl bonds (GO: 0016798).

### 3.5 Identification of key miRNA-mRNA modules by integrated omics analysis

**Figure 7:**
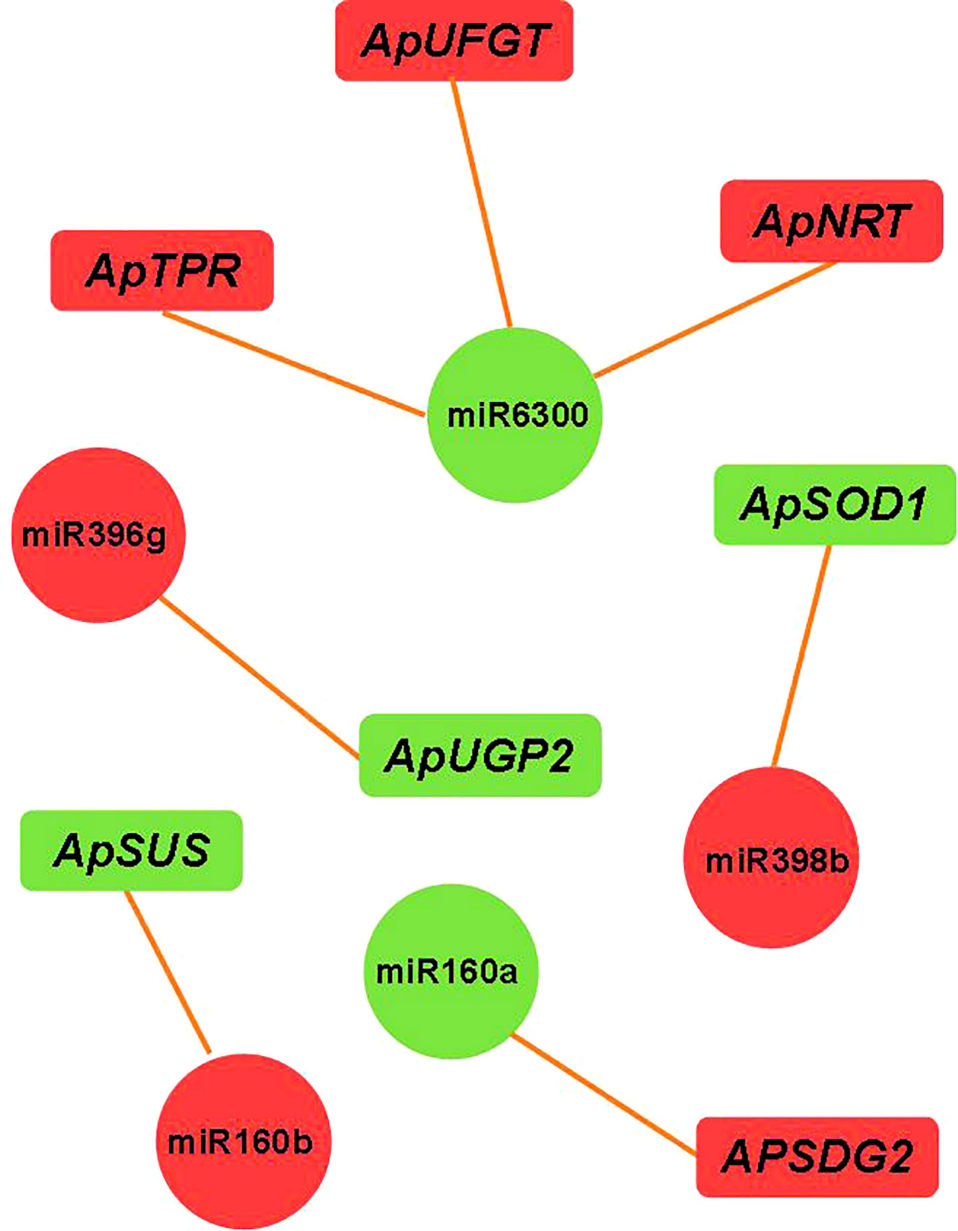
miRNA-target gene regulatory relationship network. Note Green indicates downregulated expression; red indicates upregulated expression.

**Figure 8:**
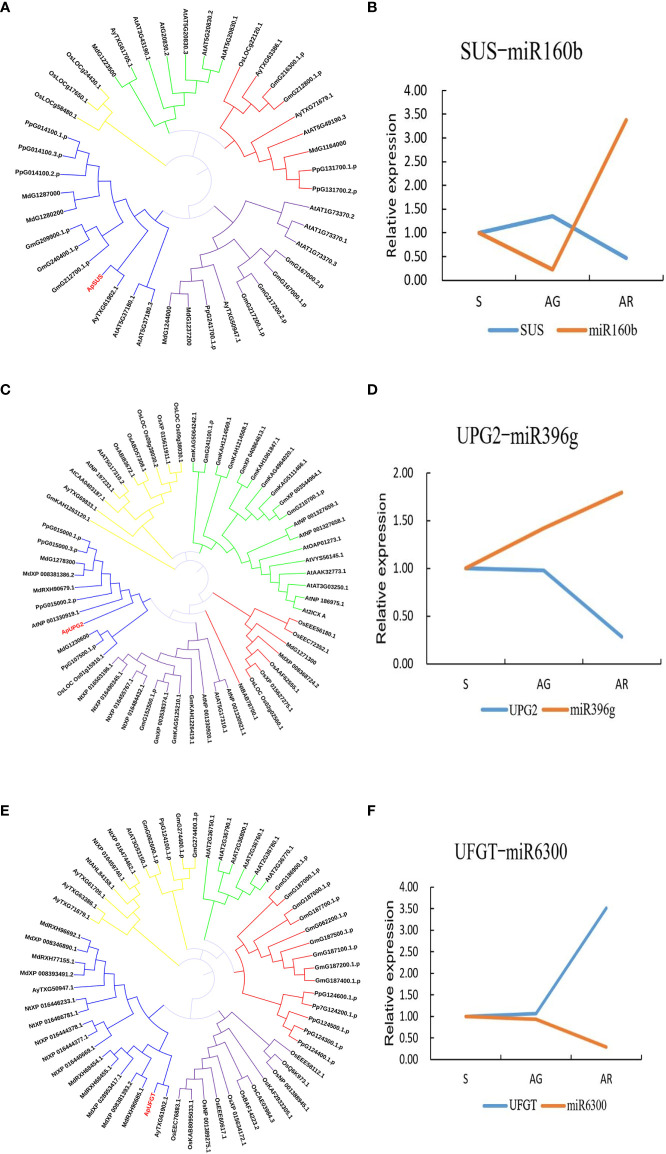
Phylogenetic tree of three key genes and fluorescence quantitative verification results of their corresponding miRNA. **(A)** Phylogenetic tree of SUS. **(B)** Relative expression of *ApSUS*-mi160b during leaf color transition. **(C)** Phylogenetic tree of UGP2.**(D)** Relative expression of *ApUPG2*-miR396g during leaf color transition. **(E)** Phylogenetic Tree of UFGT. **(F)** Relative expression of *ApUFGT*-miR6300during leaf color transition.

We constructed an miRNA-mRNA regulatory network and explored the relationship between the miRNA and its target genes. Through miRNA-mRNA association analysis, five differentially expressed modules (DEMs) were found. One miRNA targeted three genes, and four miRNAs targeted a single gene (as shown in **Figure 7**). Among them, miR6300 targeted *ApTPR* (tetratricopeptide repeat (TPR)-like superfamily protein), *ApUFGT* (UDP-glucosyl transferase), and *ApNRT* (a nitrate transporter); miR160b targeted *ApSUS* (sucrose synthase 6); and miR396g targeted *ApUGP2* (UTP—glucose-1-phosphate uridyltransferase). UFGT is the last enzyme in the process of anthocyanin synthesis. The glucose group of UDP-glucose is mainly transferred to the C3 hydroxyl group of the anthocyanin molecule, and unstable anthocyanins are transformed into stable anthocyanins. Sucrose synthase (SUS) is involved in the process of glucose metabolism, and its main function is to catalyze sucrose (D-sucrose) and UDP to synthesize fructose and UDP glucose. UGP2 is a UTP-glucose-1-phosphate uridylate transferase, and its main function is to transfer UTP (uridine triphosphate) to phosphoric acid to UDPG (uridine diphosphate glucose). We verified the expression levels of miR160b and *ApSUS*, miR396g and *ApUGP2*, and miR6300 and *ApUFGT* by quantitative real-time PCR (qRT-PCR) and found that there was a complementary relationship between the miRNA and target gene at a specific period. We speculated that there might be a targeted regulatory relationship between the miRNA and the target gene. We use phylogenetic analysis to define the different groups that exist in the plant genome. The phylogenetic tree was constructed using the amino acid sequences of ApSUS, ApUGP2, and ApUFGT of *A. p. subsp. mono* and the amino acid sequences of SUS, UGP2, and UFGT of A. thaliana, Oryza sativa, Nicotiana tabacum, Glycine max, Prunus persica, and Malus pumila (as shown in **Figure 8**). The phylogenetic tree contained all the variation sequences (Supplementary Table S3). The results showed that the statistical support for most branches was very low (data not shown).

## 4 Discussion

*Acer p. subsp*. *mono* is an important native tree species in northern China with colorful leaves and high ornamental value. In recent years, high-throughput sequencing and bioinformatics tools have been used to confirm that some miRNAs are related to leaf color changes in some plants, such as in *Koelreuteria paniculata* ‘jinye’ (Guo et al., 2022), *Anthurium andraeanum* ‘Sonate’ (Jiang et al., 2018), and *Liquidambar formosana* Hance (Wen et al., 2020). However, the regulatory mechanism of miRNA-mRNA is species-specific. To better understand the regulatory mechanism of miRNAs-mRNA and the changes in small-molecule metabolites in the leaves during color change in wild *A. p. subsp. mono* bud mutation branches, we detected the physiological indexes of the leaves of the bud mutation branches and analyzed the differential metabolites in the leaves. Combined with the differential metabolite analysis results, we further analyzed the mRNA and miRNA sequences of *A. p. subsp. mono* leaves during color and transition and used high-throughput sequencing and bioinformatics tools to identify known miRNAs, new miRNAs, and their target genes.

Since the anthocyanin synthesis pathway was also found to be a very conserved network in different plant species in previous studies (Tanaka and Ohmiya, 2008). It starts with the chalconesynthase (CHS) mediated synthesis of naringenin chalcone from 4-coumaroyl-CoA and malonyl-CoA. Then, naringenin chalconeis is omerized by chalcone isomerase (CHI) to naringenin. Flavanone 3-hydroxylase (F3H) converts naringenin into dihydrokaempferol which can be further hydroxylated by flavonoid 3’ -hydroxylase (F3’H) or flavonoid 3’,5’ -hydroxylase (F3’5’H) into other dihydroflavonols, dihydroquercetin and dihydrotricetin, respectively. Then, the dihydroflavonols are converted into colorless leucoanthocyanidins by dihydroflavonol 4-reductase (DFR) and subsequently to colored anthocyanidins by anthocyanidin synthase(ANS). Anthocyanidins are glycolsylated to facilitate their accumulation in cells by the enzyme flavonoid 3-O-glucosyltransferase (UFGT), and might be further acylated with aromatic acyl groups by acyltransferases (Li et al., 2018; Guo et al., 2019). The activities of the antioxidant enzymes SOD, POD, CAT, and PAL in the autumn red leaves were higher than those in the autumn green leaves. Naringenin chalcone, naringenin, and dihydroquercetin—the intermediate products of the main pathway of anthocyanin synthesis—accumulated significantly, and the main pathways of anthocyanin synthesis were activated. The catalytic reaction was towards the synthesis of cyanidins, and cyanidins were the main pigments in the red leaves of *A. p. subsp. mono.* In addition, the content of soluble sugar in the red leaves of the bud mutation branches of *A. p. subsp. mono* was low. Through further metabonomics analysis, we found that D-sucrose in the red leaves also decreased significantly, and anthocyanin accumulation was negatively correlated with decreased soluble sugar. At the same time, the UDP content in the red leaves increased, the UDPG content decreased, and fructose did not change significantly. D-Sucrose and UDP synthesize fructose and UDPG under the catalysis of SUS. A decrease in photosynthetic pigment content reduced the accumulation of D-sucrose *in vivo*, and UDPG also consumed D-sucrose in the leaves. A large amount of uridine diphosphate glucose (UDPG) was consumed during the conversion of unstable anthocyanins into stable anthocyanins. Therefore, we hypothesize that the leaves of bud mutation branches are more sensitive to the perception of the external environment. Under the influence of environmental factors such as decreasing temperature and light intensity in autumn, the upstream genes regulating the anthocyanin synthesis pathway in the leaves of bud mutation branches were activated earlier and were heavily transcribed and translated, which promoted the synthesis of anthocyanins and thus led to the reddening of the leaves of bud mutation branches.

The changes of anthocyanin content in plants are closely related to the expression status of key genes of the anthocyanin synthesis pathway. Among the early biosynthesis genes include chalcone synthase (*CHS*),chalcone isomerase (*CHI*), and flavanone-3-hydroxylase (*F3H*), while others are classified as late biosynthesis genes, including dihydroflavonol-4-reductase (*DFR*), anthocyanidin synthase (*ANS*), and UDP-flavonoid glucosyl transferase (*UFGT*) (Lloyd et al., 2017). We predicted that miR160b targeted *ApSUS*, miR396g targeted *ApUGP2*, and the expression levels of miR160b and miR396g were upregulated in the red leaves of the bud mutation branches of *A. p. subsp. mono*. UGP2 is a UTP-glucose-1-phosphate uridylate transferase, the main function of which is to catalyze UTP to UDPG, and UDPG is the substrate in the process of transforming unstable anthocyanins into stable anthocyanins. UFGT, as the last enzyme of the anthocyanin synthesis pathway, plays an important role in anthocyanin accumulation (He et al., 2019), mainly transferring the glucose group on UDPG to the C3 hydroxyl group of the anthocyanin molecule and transforming unstable anthocyanins into stable anthocyanins. The decrease in UDPG content with the change in leaf color also shows that our speculation was correct, which is also consistent with previous findings (Zhao et al., 2012). We predicted that miR6300 targets UFGT. The expression of *ApUFGT* was upregulated with the downregulation of miR6300, and so the content of cyanidin-3-*O*-glucoside in the red leaves was significantly higher than that in the green leaves. This is also consistent with our GO classification of differentially expressed mRNAs, which were significantly enriched in photosynthesis (GO: 0015979), carbohydrate metabolic process (GO: 0005975), and disaccharide metabolic process (GO: 0005984) and pathways of photosynthesis, glucose metabolism, and glycosyl hydrolysis. Anthocyanins accumulated, chlorophyll degraded, anthocyanin/chlorophyll values increases, and the pentagonal maple leaves turned red (Weng et al., 2020), which is consistent with the discoloration mechanism of many purple-red leaves. In the discoloration reaction dominated by anthocyanins, the color difference is related to the anthocyanin content, and the different glycoside types combined with the same anthocyanins will also lead to differences in leaf color (Matthews et al., 2003). In *A. p. subsp. mono*, the glycoside types of cyanidin, pelargonidin, malvidin, petunidin, delphinidin, and peonidin were detected, among which the glycoside types of cyanidin accounted for a relatively high proportion. Two glycoside types of cyanidin, namely cyanidin 3-*O*-glucoside and cyanidin 3-*O*-galactoside, were detected simultaneously. Carotenoids, which are also an auxiliary pigment of plant photosynthesis (Ge et al., 2011), regulate yellow leaf color in plants. In this experiment, carotenoids accumulated when the five buds changed into branches and the leaves turned red. In addition, the contents of ascorbic acid and jasmonic acid in the red leaves were significantly lower than those in the green leaves of the wild type. The significant upregulation of ABA and IBA in the red leaves also indicated that the leaves senesced and transitioned into the abscission stage.

**Figure 9:**
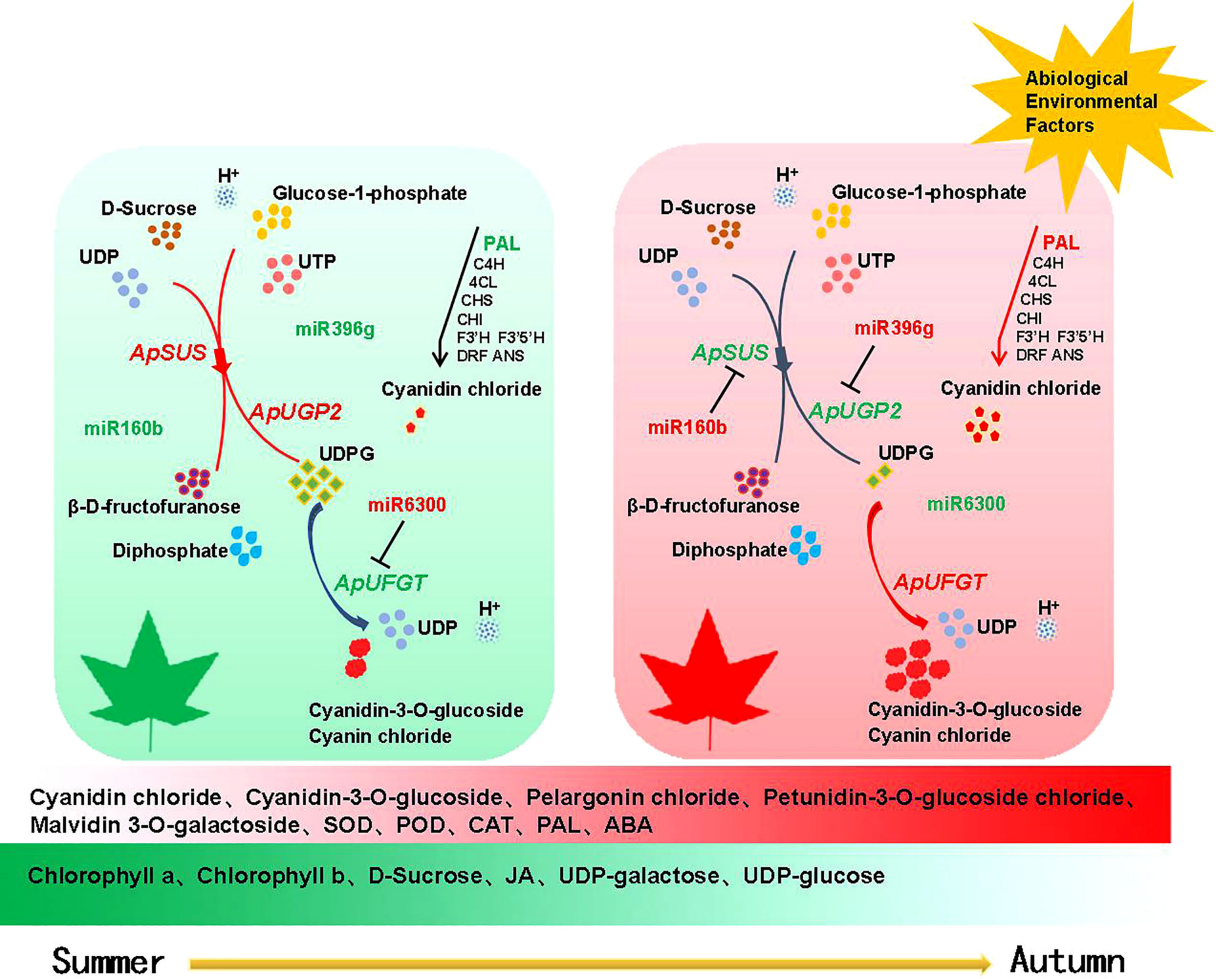
Regulation pattern of miRNAs and target genes during color transition in *A. p. subsp. mono* leaves. Note Red represents upregulated expression and green represents downregulated expression.

In previous studies on miR6300, miR160b, and miR396g, it was found (Liu et al., 2021) that miR6300 targeted *ANS* in the honeysuckle anthocyanin metabolic pathway, while another study (Zhu, 2019) found that miR6300 had a significant correlation with the key genes of the mevalonate metabolic pathway. He et al. found that miR6300 targeted *UFGT* in sweet potato, thus regulating anthocyanin biosynthesis in sweet potato (He et al., 2019), while Sun (Sun, 2017) and others found that miR160 participates in the response of plants to adversity. miR396g targets *WD40* and then regulates anthocyanin synthesis in sweet potato root tuber (Huang et al., 2019). Li et al. found that the miR396g gene targeting chloroplast development and hormone metabolism in *Ginkgo biloba* leaves regulates leaf color (Li et al., 2019). We predicted through correlation analysis that miR160b targeted *ApSUS*, miR396g targeted *ApUGP2*, and miR6300 targeted *ApUFGT*. Fluorescence quantitative PCR, differential metabolite analysis, and various physiological indexes further supported the rationality of the above inference. We synthesized the physiological response model of leaf color change in *A. p. subsp. mono* (as shown in **Figure 9**) by detecting physiological indexes, analyzing differential metabolites, and sequencing the transcriptome and miRNA results.

In summary, as an important factor of post-transcriptional regulation, the role of miRNA in the color transition of *A. p. subsp. mono* leaves is rarely reported. In this study, the expression patterns of miRNAs during the color transition period of *A. p. subsp. mono* leaves were analyzed, and the miRNA-mRNA regulatory modules in the color transition process of *A. p. subsp. mono* leaves were demonstrated for the first time. The experimental results provide a foundation for improving *A. p. subsp. Mono*. Our next work focuses on constructing genetic transformation system for *A. p. subsp. mono.*, and validates the targeting relationships of miR160b-*ApSUS*, miR396g-*ApUGP2*, and miR6300-*ApUFGT*. We hope to further explore the molecular regulatory mechanism of leaf color change in *A. p. subsp. mono.*

## Data availability statement

The datasets presented in this study can be found in online repositories. The names of the repository/repositories and accession number(s) can be found below: https://www.ncbi.nlm.nih.gov/geo /, GSE212656; https://www.ncbi.nlm.nih.gov/geo/ , GSE212815.

## Author contributions

JC conceived and designed the experiments and performed the sample collection. HM collected some samples. BL analyzed the data and wrote the paper. KZ analyzed the data too. All authors have read and approved the manuscript.
