# Supplementary figures and images for: Regulatory mechanisms and metabolic changes of miRNA during leaf color change in the bud mutation branches of Acer pictum subsp. mono
Source: Front Plant Sci. 2023 Jan 12;13:1047452. doi: 10.3389/fpls.2022.1047452 (PMC9879609; doi:10.3389/fpls.2022.1047452)

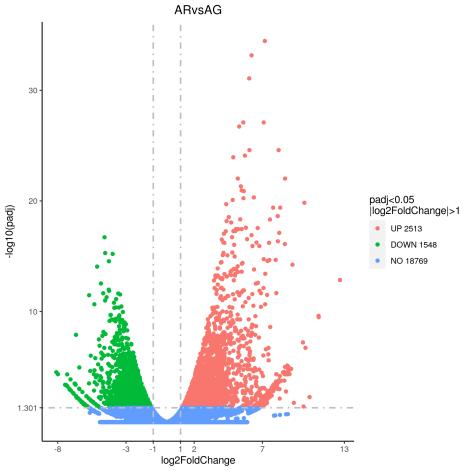

Supplement: Supplementary file 4 [file Image_1.jpeg]

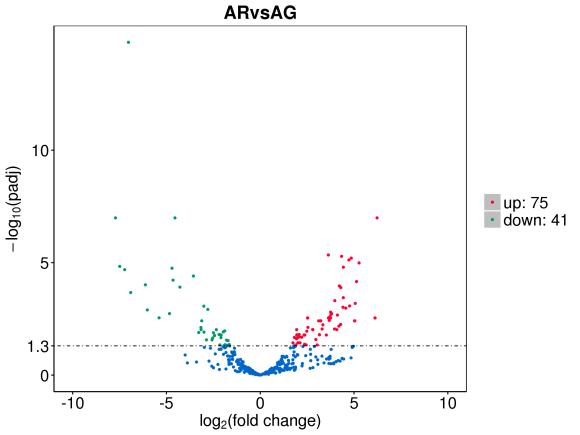

Supplement: Supplementary file 5 [file Image_2.jpeg]
